# Combined Effects of *Eurycoma longifolia* and Testosterone on Androgen-Deficient Osteoporosis in a Male Rat Model

**DOI:** 10.1155/2012/872406

**Published:** 2012-08-09

**Authors:** Halimatun Saadiah Abdul Razak, Ahmad Nazrun Shuid, Isa Naina Mohamed

**Affiliations:** ^1^Department of Biomedical Sciences, Faculty of Health Sciences, National University of Malaysia (Universiti Kebangsaan Malaysia), 50300 Kuala Lumpur, Malaysia; ^2^Department of Pharmacology, Faculty of Medicine, National University of Malaysia (Universiti Kebangsaan Malaysia), 50300 Kuala Lumpur, Malaysia

## Abstract

Androgen-deficient osteoporosis in men is treated with testosterone therapy, which is associated with side effects. *Eurycoma longifolia* (EL) is known to possess androgenic properties and has been reported to protect bone from androgen-deficient osteoporosis in experimental animal models. The present study aimed to determine the effectiveness of combination therapy of EL and testosterone (T) in treating androgen-deficient osteoporosis. Forty male Sprague-Dawley rats were divided into: sham-operated (SHAM), orchidectomized-control (ORX), orchidectomized with testosterone (ORX + T), orchidectomized with EL (ORX + EL), and orchidectomized with combined T and EL therapy (ORX + T + EL). EL was administered via oral gavages daily at the dose of 15 mg/kg. T was injected intramuscularly at 8 mg/kg and 4 mg/kg for the ORX + T and ORX + T + EL groups, respectively. Following 6 weeks of treatment, the osteocalcin levels of ORX + T and ORX + T + EL groups were significantly lower than the SHAM group (*P* < 0.05). The posttreatment CTX levels of ORX + T and ORX + T + EL groups were significantly lower than their pretreatment levels (*P* < 0.05). Biomechanically, the strain parameter of the ORX + T + EL group was significantly higher than the ORX group (*P* < 0.05). Thus, the combination therapy of EL and low-dose T has potential for treatment of androgen-deficient osteoporosis. The lower T dose is beneficial in reducing the sideeffects of testosterone therapy.

## 1. Introduction

Partial androgen deficiency, which is common in aged men, may lead to bone loss and osteoporosis [1]. Androgen deficiency due to natural aging is the main cause of osteoporosis in men [2]. This bone-thinning disease was given more attention lately after becoming one of the main causes of morbidity and mortality in older men. In the United States, 1.5 million men over 65 years old were reported to suffer from osteoporosis [3]. One of the complications of osteoporosis is pathological fracture when the osteoporotic bone breaks with minimal force. Men are more likely than women to die following a hip fracture [4], and it is estimated that 31% of men with hip fracture died within a year after fracture [5].

Among the accepted treatment for androgen-deficient osteoporosis in men is testosterone replacement therapy (TRT). Studies have shown that TRT increased bone mass [6] and improved muscle strength and mass in testosterone-deficient male [7, 8]. It can be given via various routes, but intramuscular injection is widely used as it provides immediate testosterone surge. This form of administration is painful and associated with prostate cancer and increased haematocrit [9]. Other routes of testosterone have their disadvantages such as liver tumors with the oral form or transference to women and children by skin contact with the transdermal form. 

*Eurycoma longifolia* (EL), classified under the *Simaroubaceae* family, is a traditional medicinal plant, known as “*Pasak Bumi*” in Indonesia, “*Cay Ba Binh*” in Vietnam, “Langir Siam” in Bahrain, and “Tung Sawa” in Thailand [10]. EL is a herbaceous plant that grows slowly. It can reach a maximum height of 15 to18 meters and bear fruit after 2 to 3 years. Initially, the fruits are green in colour but turned dark red when ripe. The leaves are oval in shape with 10 to 30 leaflets and arranged spirally. EL was officially announced as a protected plant in many growing areas, including Malaysia [11].

It was reported that EL has aphrodisiac effects and the ability to increase the testosterone levels [12]. Supplementation of EL to male with idiopathic infertility improved their sperm concentration, motility, and morphology [13]. Due to its androgenic effects, EL may be useful in the treatment of diseases related to androgen deficiency. Recently, a study demonstrated that EL has potential in the treatment of osteoporosis due to androgen deficiency in a rat model [14]. Both EL and testosterone were found to be capable of preventing bone calcium loss in orchidectomized rats [14]. The dose of EL used in the present study was 15 mg/kg rat weight, which was very much lower than its lethal dose 50 (LD_50_) of more than 5000 mg/kg [15]. Therefore, EL can be combined with testosterone to lower the testosterone dose, thus minimizing side effects. 

The aim of the study was to determine the effects of the combination therapy of EL and low-dose testosterone on bone markers and bone strength of orchidectomized rats. Their effects on bone were compared to a group of orchidectomised rats receiving the full dose of testosterone alone. Aged orchidectomized rat is an accepted model for androgen-deficient osteoporosis [16]. The 6-week duration of treatment in the present study was similar to an earlier study [14] and should be sufficient for orchidectomy to produce significant bone changes.

## 2. Material and Methods

### 2.1. Animal Model

Forty aged male Sprague-Dawley rats (aged 10 to 12 months) were obtained from the UKM Animal House. The rats were housed at two per cage with the surrounding temperature of 29 ± 3°C and under natural day/night cycle. They were fed commercial food pellets and tap water *ad libitum*. They were allowed to adjust to the new environment for a week before the study was started. The study was approved by the UKM Animals Ethics Committee (PP/FAR/2010/NAZRUN/14-JULY/310-JULY-2010-JUNE-2011). The rats were divided into groups of sham-operated (SHAM), orchidectomized-control (ORX), orchidectomized and given EL (ORX + EL), orchidectomized and given testosterone (ORX + T), and orchidectomised and given combined testosterone and low-dose EL (ORX + T + EL). Body weight was measured before treatment and then weekly until the end of the study. 

### 2.2. Orchidectomy Procedure

Before performing orchidectomy, the rats were anesthetized with Ketamil : Xylazil (1 : 1). A 2 cm ventral midline incision was made in the scrotum, and the skin was retracted to expose the tunica. The tunica was pierced, and the testis was pushed out and raised to expose the underlying blood vessels and tubules. The spermatic cord was clamped and tied with absorbable catgut suture at the confluence of the blood vessels and epididymis. The testis was removed, and all deferential vessels and ducts were replaced back into the tunica. Similar procedures were repeated on the other testis.

### 2.3. *Eurycoma longifolia* and Testosterone

*Eurycoma longifolia *was supplied by Phytes Biotek Sdn. Bhd. (Selangor, Malaysia), a licensed GMP manufacturer of herbal products, in the form of a freeze-dried standardized extract (batch No: TA 071210). It was extracted from the roots using a patented high-pressure water extraction process (US 7,132,117 B2), filtered at 1–4 microns and freeze-dried without maltodextrins or lactose. Physically, it was a light brown fine powder with 4–6% moisture content. Its major chemical components were proteins (31.75%), glycosaponins (41.08%), and eurycomanone (1.60%). This extract was the same form used for human consumption as health supplements.

EL aqueous extract powder was dissolved in deionized water, and 15.0 mg/kg was given to rats in the ORX + EL and ORX + T + EL groups via daily oral gavages at 9 am for 6 weeks [17]. Testosterone was purchased from TCI UK Ltd (UK). It was diluted in olive oil (Bertolli, Italy) and 8.0 mg/kg was injected intramuscularly once daily at 9 am for 6 weeks for the ORX + T group [18]. As for the low-dose T in the ORX + T + EL group, half of the dose (4.0 mg/kg) was injected once daily at 9 am for 6 weeks. The SHAM, ORX, ORX + T, and ORX + T + EL groups were also given oral gavages of vehicle (deionised water). In addition, the SHAM, ORX, and ORX + EL groups received intramuscular injections of vehicle (olive oil). Blood samples were collected before the start of treatment and after 6 weeks of treatment from retroorbital sinus after anesthetizing the rats with ether. After 3 to 4 hours, the blood was centrifuged at 3000 rpm for 10 minutes, and the serum stored at temperature of −70°C.

### 2.4. Parameters

At the end of the treatment, the rats were sacrificed with overdoses of ether, and the femora were dissected out and cleansed of all soft tissues. The femora were wrapped with gauze soaked with phosphate buffered solution, wrapped again with aluminium foil and placed in −70°C. The study groups were numbered to blind the operators. Each femur was placed on the Instron machine (Instron Microtester 5848, Instron Corp., USA) in a three-point bending configuration. The load was applied at the mid-diaphysis in an anteroposterior direction with a loading speed of 5 mm/min until the femur fractured. The load, stress, and strain-deflection curves were automatically calculated by the computer using the Bluehill software. The femora were kept moist at all times during the testing. The parameters measured were load, stress, strain, and Young's Modulus.

Bone formation marker (serum osteocalcin) and bone resorption marker (C-terminal telopeptide of type I collagen, CTX) were measured before and after treatment using ELISA technique with ELISA reader (VERSA MAX, Sunnyvale,USA). The kits used were the Rat Osteocalcin ELISA (Biomedical Technologies, Herlev, Denmark) and RatlapsTM ELISA CTX-1 kits (Nordic Biosciences, IDS, UK).

### 2.5. Statistical Analysis

For normally distributed data, the statistical test used was ANOVA followed by Tukey's HSD. Data that was not normally distributed was analyzed using Mann-Whitney followed by Kruskal-Wallis test if more than two groups were compared. The level of significance was taken as *P* < 0.05.

## 3. Results

**Figure 1:**
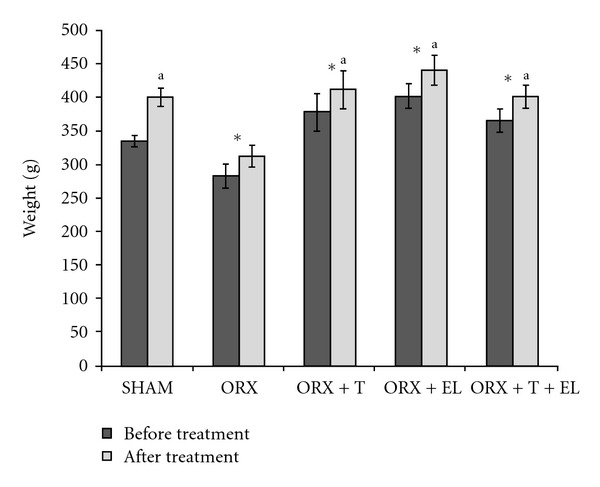
Mean body weight before and after treatment. Data presented as mean ± SEM. SHAM: sham-operated control. ORX: orchidectomized control. ORX + T: orchidectomized supplemented with testosterone. ORX + EL: orchidectomized supplemented with EL. ORX + T + EL: Orchidectomized and combination treatment of testosterone with EL. *Significant difference before and after treatment within the group (*P* < 0.05). ^a^Significant difference after treatment compared to ORX group (*P* < 0.05).

There were no significant differences in the mean body weight for all the groups of rats at the start of the study. At the end of the study, all groups except the SHAM group showed significantly higher body weight compared to their pretreatment weights. The posttreatment weight of the ORX group was significantly lower than the rest of the groups (Figure 1).

**Figure 2:**
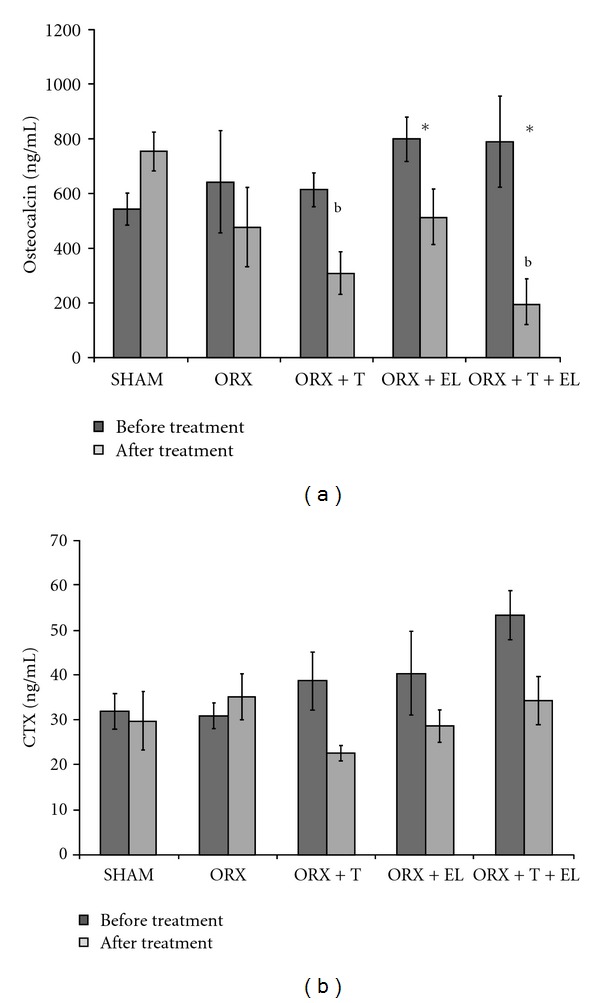
(a) Mean serum osteocalcin levels for all the groups before and after treatment. (b) Mean serum C-terminal telopeptide of type I collagen for all the groups before and after treatment. Data presented as mean ± SEM. SHAM: sham-operated control. ORX: orchidectomized control. ORX + T: orchidectomized supplemented with testosterone. ORX + EL: orchidectomized supplemented with EL. ORX + T + EL: orchidectomized and combination treatment of testosterone with EL. ^b^the posttreatment level is significant different compared to the posttreatment level of SHAM group (*P* < 0.05). *Significant difference before and after treatment in each group (*P* < 0.05).

There was no significant difference in the osteocalcin levels between the SHAM and ORX groups. The posttreatment osteocalcin levels of ORX + EL and ORX + T + EL groups were significantly lower compared to their pretreatment levels. The posttreatment osteocalcin levels of ORX + T and ORX + T + EL groups were also significantly lower compared to the posttreatment level of the SHAM group. There was also no significant difference in the pre- or posttreatment osteocalcin levels of the ORX + T + EL group compared to the ORX + T or ORX + EL group (Figure 2(a)). 

There was no significant difference in the CTX levels between the SHAM and ORX groups. The posttreatment CTX levels of ORX + T and ORX + T + EL groups were significantly lower than their pretreatment levels. The posttreatment CTX level of ORX + EL group also appeared to be lower than its pre-treatment level, but the difference was not significant. There was no significant difference in the pre- or posttreatment CTX levels of the ORX + T + EL group compared to the ORX + T or ORX + EL group (Figure 2(b)). 

**Figure 3:**
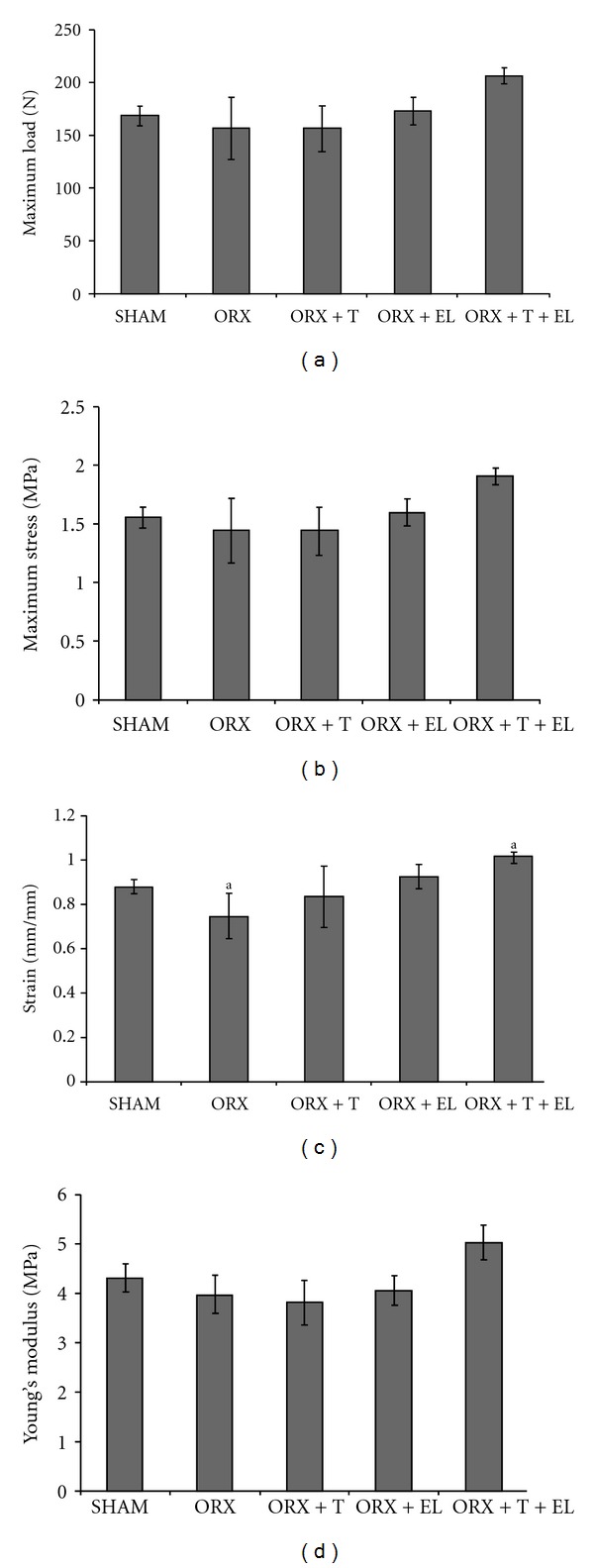
(a) Maximum load values for all the groups. (b) Maximum stress values for all the groups. (c) Strain values for all the groups. ^a^Significant difference between the groups (*P* < 0.05). (d) Young's modulus for all the groups. Data presented as mean ± SEM. SHAM: sham-operated control. ORX: orchidectomized control. ORX + T: orchidectomized supplemented with testosterone. ORX + EL: orchidectomized supplemented with EL. ORX + T + EL: Orchidectomized and combination treatment of testosterone with EL.

As for the biomechanical parameters, there was no significant difference between the SHAM and ORX groups, although these parameters appeared to be lowered in the ORX group. There was no other significant difference between the groups except for the significantly higher strain parameter for the ORX + T + EL group compared to the ORX group. The ORX + T + EL group also had the highest value for the rest of the biomechanical parameters but were not statistically significant compared to the other treatment groups (Figures 3(a), 3(b), 3(c), and 3(d)).

## 4. Discussion

All the rats in the study had gained weight, but the orchidectomised rats failed to gain weight normally, resulting in their body weight to be significantly lower than the sham-operated rats at the end of the study. Testosterone reduction due to orchidectomy may have caused the weight loss due to loss of muscle and bone mass [19]. Testosterone, EL, or combination of both treatments could maintain the weight gain of orchidectomised rats. Testosterone has been shown to stimulate the release of growth hormone [20] which then stimulates the expression of insulin-like growth factor-1 (IGF-1). IGF-1 has anabolic effects on skeletal muscle mass, stimulates protein synthesis, decreases protein degradation, and increases the lean body mass [21–23]. EL supplementation has been found to increase the body mass index [24] and has ergogenic effects [25] in adult male subjects. Based on the findings of these studies, it is not surprising to find that the combination of EL and low-dose testosterone was able to maintain the weight gain in orchidectomised rats.

In normal aged rats, the growth plate should have stabilized resulting in balanced bone formation and resorption [26] as seen in the sham-operated rats used in this study. The bone formation (osteocalcin) and resorption (CTX) markers of the sham-operated rats were not significantly different before and after treatment. Theoretically, there should be increased bone markers with the high bone turnover due to gonadectomy in aged male rats [27]. However, this was not observed in the present study. In comparison with the findings of a previous study [14], there was also no significant change in the osteocalcin level of orchidectomised rats, but the CTX level was not similarly raised although the trend was there.

Treatment with testosterone or EL significantly lowered the posttreatment levels of osteocalcin and CTX. Studies have shown that testosterone treatment prevented cancellous bone loss in orchidectomised rats, similar to the action of estrogen on bone resorption [28, 29]. The findings of the present study were consistent with an earlier study by Shuid et al. [14] that EL was capable of preventing bone loss in orchidectomised rats. Combination of testosterone and EL was able to significantly lower both the formation and resorption markers. This meant that the combination treatment could reduce bone turnover in orchidectomised rats, much better than treatment with EL or testosterone alone. Combination of EL and testosterone seemed to produce synergistic effects in maintaining the bone turnover in orchidectomised rats.

The osteocalcin or CTX levels of the combination of EL and testosterone group were not significantly different compared to the testosterone or EL group. When compared to the pretreatment levels, combination of EL and testosterone has resulted in significantly lower osteocalcin or CTX levels. Treatment with testosterone or EL was only able to lower either one of the bone markers. This indicated that combination treatment could reduce bone turnover in orchidectomised rats, much better than treatment with EL or testosterone alone. This is beneficial as high bone turnover can lead to thinning of the bone, resulting in abnormal bone microarchitecture and reduced bone mineralization [30–32]. Combination of EL and testosterone seemed to produce synergistic effects in reducing the bone turnover of orchidectomised rats.

Shuid et al. [14] reported that reduction in bone calcium can be recovered with EL or testosterone treatment. To the best of our knowledge, the effects of EL on bone biomechanical testing were never reported. In the present study, there was no significant difference in the bone biomechanical strength of the orchidectomised rat compared to the sham-operated rats. Other studies have also found no significant change in the bone biomechanical strength of orchidectomised rats [33, 34]. The bone strength is determined by the bone mass and the intrinsic properties of the bone material [35, 36]. This indicated that the bone changes induced by orchidectomy may not be sufficient to affect bone strength. The bone strength of rats that have received the combination of EL and testosterone was not different from rats receiving testosterone or EL alone. However, these rats have recorded a significantly higher strain parameter than the orchidectomised rats. This meant that the femora of rats receiving the combination of EL and testosterone could resist deformation with the force applied, much better than the other groups. Treatment with EL also seemed to produce higher biomechanical parameters although they did not reach statistical significance. The combination of EL and testosterone has resulted in the highest values for all the biomechanical parameters but only the strain parameter reached statistical significance. This meant that the combination of EL and testosterone was synergistic in maintaining the bone strength against the deleterious effects of orchidectomy.

The combination of EL and testosterone was able to reduce bone turnover and improve the bone strength of orchidectomised rats more effectively than treatment with EL or testosterone alone. It is important to note that the dose of testosterone used in combination with EL was only half of that used in the ORX + T group. Thus,combination of EL and lowdose testosterone was synergistic in maintaining the bone turnover and strength of orchidectomised rats.

## 5. Conclusion 

Combination of EL and testosterone has shown potential for the treatment of androgen-deficient osteoporosis. With this combination, lower dose of testosterone can be used, therefore minimizing its side effects. This opens the door for future studies to explore the possible role of combination therapy in the treatment of androgen-deficient osteoporosis in men.
